# Academic burnout under geopolitical and economic uncertainty: longitudinal associations with future development anxiety, employment competition pressure, and perceived structural university support

**DOI:** 10.3389/fpsyg.2026.1951855

**Published:** 2026-09-28

**Authors:** Qingzhe Zhang

**Affiliations:** School of Marxism, North China University of Water Resources and Electric Power, Zhengzhou, China

**Keywords:** academic burnout, employment competition pressure, future development anxiety, geopolitical and economic uncertainty, perceived structural university support

## Abstract

**Introduction:**

Students may interpret geopolitical and economic change as a threat to future development, but its cross-wave associations with academic burnout remain unclear.

**Methods:**

This study analyzed a two-wave convenience sample from four universities in Zhengzhou, China. The baseline sample comprised 1,823 students, of whom 1,215 completed matched follow-up after approximately 5 months. Wave-specific factor analyses, covariance-based confirmatory factor analysis, partial least squares structural equation modeling, and covariance-based sensitivity models were used.

**Results:**

After baseline adjustment, T1 perceived geopolitical and economic uncertainty was positively associated with T2 future development anxiety (beta = 0.175, *P* < 0.001) and T2 academic burnout (beta = 0.148, *P* < 0.001). T1 future development anxiety was positively associated with T2 academic burnout (beta = 0.176, *P* < 0.001), and the reverse cross-wave association was weaker (beta = 0.087, *P* < 0.001). The prospective-concurrent indirect association through T2 future development anxiety was significant (beta = 0.037, *P* < 0.001), but the mediator and outcome were measured concurrently. Employment competition pressure strengthened the focal association (beta = 0.105, *P* < 0.001), whereas perceived structural university support attenuated this moderation (three-way interaction beta = −0.114, *P* < 0.001).

**Discussion:**

The results indicate small to moderate cross-wave between-person associations. They do not establish within-person causation or temporal mediation. Universities may use these patterns to inform targeted support, but intervention effectiveness requires direct evaluation.

## 1 Introduction

Academic burnout is a negative learning state that develops under sustained learning demands and academic pressure. It is typically manifested in emotional and physical exhaustion, detachment from learning activities, and reduced academic efficacy. Among university students, academic burnout is associated not only with lower learning engagement and academic performance but may also undermine psychological adjustment and preparedness for future development (Salmela-Aro et al., 2009; Schaufeli et al., 2002). Against a backdrop in which geopolitical conflicts, fluctuations in economic growth, industrial restructuring, and changes in the employment environment are intertwined, university students' learning may no longer be shaped solely by campus-based factors such as coursework and examinations; it may increasingly be embedded in their judgments of the broader development environment. When students are uncertain whether their current investment in learning can translate into stable employment opportunities and developmental returns, their learning goals, willingness to engage, and sense of efficacy may all be affected. Identifying the sources of risk for academic burnout and the conditions amenable to intervention therefore remains an important task in higher education research.

Existing research has primarily explained academic burnout among university students in terms of course workload, examination pressure, family financial strain, employment pressure, and social support (Bakker and Mostert, 2024; Kong and Zeng, 2023; Liu et al., 2023; Qiang et al., 2024; Zhang et al., 2025). These studies have provided useful explanations of how campus-based and individual stressors relate to academic burnout, but they have paid less attention to how university students interpret a continuously changing external environment. Overall, existing research has generally treated academic burnout as the joint outcome of internal campus demands, individual psychological characteristics, and direct life stress, while giving comparatively limited consideration to the penetration of the macro-level political and economic environment into students‘ learning. Armed conflicts, fluctuations in energy and trade, restrictions on cross-border mobility, and changes in expectations regarding industrial development may alter students' judgments about employment, further study, and career opportunities. Previous studies have shown that university students‘ perceived environmental uncertainty is associated with higher anxiety and lower academic engagement, whereas negative responses to uncertainty are also associated with higher academic burnout (Kong and Zeng, 2023; Qiang et al., 2024). External events do not necessarily lead to academic burnout in themselves; how students appraise the relationship between these events and their personal goals, existing resources, and future development may be a key process through which the external environment affects their learning state (Lazarus and Folkman, 1984). It is therefore necessary to shift the analytical focus from objective macro-level events to students' subjective risk appraisals of changes in the broader environment and to examine whether such appraisals are associated with a further deterioration in their current learning state.

This study defines university students' judgments concerning the unpredictability and uncontrollability of international conflicts, fluctuations in energy prices, changes in trade and external demand, instability in cross-border mobility, and the consequences of these developments for their personal futures as perceived geopolitical and economic uncertainty. This construct reflects university students' subjective appraisal of changes in the external environment and their implications for personal development. It is not equivalent to objective geopolitical risk or macroeconomic volatility; rather, it emphasizes whether students interpret such changes as realistic risks that may affect their employment, further study, career choices, and life stability. Cognitive appraisal theory of stress proposes that stress responses do not depend entirely on external events themselves but on how individuals appraise the relationship between those events and their goals, resources, and coping capacities (Lazarus and Folkman, 1984). When university students believe that geopolitical and economic changes may affect employment opportunities, further-study choices, and life stability, their perceptions of future predictability and personal control may decline. Uncertain circumstances may also heighten attention to potential threats and expectations of negative outcomes, thereby increasing persistent worry (Grupe and Nitschke, 2013). At the same time, uncertainty in the macro-level environment may weaken students‘ stable expectations regarding the relationship between academic investment and developmental returns, leading them to question whether their current efforts can produce the expected educational and occupational benefits. Persistent attention to risk may not only consume cognitive and emotional resources but may also induce reduced learning engagement and psychological detachment. Previous research has found that university students' perceived environmental uncertainty is associated with higher anxiety and lower academic engagement (Kong and Zeng, 2023). Perceived geopolitical and economic uncertainty can therefore be regarded as an external stressor that extends beyond campus boundaries and may be prospectively associated with subsequent academic burnout.

Future development anxiety may be one psychological link between perceived geopolitical and economic uncertainty and academic burnout. Future anxiety concerns worry, fear, and threat when individuals contemplate an uncertain future (Zaleski, 1996). Conservation of resources theory proposes that anticipated resource loss can consume attention, emotion-regulation capacity, and self-control (Hobfoll, 1989). Students who worry about employment, competition, or disrupted plans may therefore have fewer resources available for current academic tasks (Qiang et al., 2024; Tan et al., 2026). Academic exhaustion and reduced efficacy may also coincide with less favorable assessments of future competitiveness (Schaufeli et al., 2002). These reciprocal associations are theoretically plausible, but the present design cannot establish a within-person mechanism.

The association between perceived geopolitical and economic uncertainty and academic burnout may also depend on students' developmental environment. Employment competition pressure may make students more likely to interpret changes in the external environment as direct threats to career development. When students perceive that job opportunities are limited, employment competition is intense, or their own competitive advantages are insufficient, macro-level uncertainty that might otherwise seem relatively distant becomes more concrete and directly relevant to their personal development interests. Previous research has found that university students' perceptions of declining employment opportunities are associated with greater employment pressure and that external employment support can alleviate such pressure (Yang et al., 2022). Under these conditions, students may be more likely to believe that changes in the external environment will constrain their development opportunities and reduce their confidence in the returns to their current learning investment; concerns arising from geopolitical and economic uncertainty may then be more readily translated into academic burnout. By contrast, academic guidance, employment services, psychological support, information resources, and development opportunities provided by universities may help students form clearer judgments about their future development and reduce the perceived threat of external change. Related research indicates that teacher support, development opportunities, and university resources can help students cope with academic demands and reduce the risk of academic burnout (Bakker and Mostert, 2024). Social cognitive career theory emphasizes that environmental barriers and support resources influence career adaptation and developmental choices (Lent et al., 2000), whereas the stress-buffering model of social support proposes that supportive resources can reduce the adverse effects of stressful events on individuals' psychological states and behavioral functioning (Cohen and Wills, 1985).

The four theoretical perspectives serve distinct functions. Cognitive appraisal theory explains how external change becomes a personally relevant threat. Conservation of resources theory explains why sustained worry may coincide with depleted academic functioning. Social cognitive career theory identifies career barriers and institutional supports that shape development-related appraisals. Stress-buffering theory motivates the prediction that support attenuates a stress-related association. This division of labor connects the predictor, indirect association, and interaction hypotheses without treating the theories as interchangeable.

Accordingly, this two-wave study addressed three questions using baseline-adjusted autoregressive and cross-lagged paths, bootstrap tests of indirect associations, and interaction analyses. First, was T1 perceived geopolitical and economic uncertainty associated with T2 future development anxiety and academic burnout? Second, were FDA and academic burnout reciprocally associated across waves, and was PGECU indirectly associated with T2 burnout through concurrent T2 FDA? Third, did ECP strengthen the focal association, and did PSUS attenuate that moderation? Concurrent T1 paths were estimated only as extended descriptive associations.

## 2 Theoretical foundations and hypothesis development

### 2.1 Prospective associations of perceived geopolitical and economic uncertainty

Cognitive appraisal theory of stress proposes that individuals‘ judgments of external events shape their stress responses (Lazarus and Folkman, 1984). When students believe that changes in the external environment may affect employment, further study, and career development, it becomes more difficult for them to form stable expectations about the future. The less predictable and controllable the environment appears, the more likely individuals are to attend to potential risks and worry about possible adverse outcomes (Carleton et al., 2007; Freeston et al., 1994; Grupe and Nitschke, 2013; Kong and Zeng, 2023). Higher perceived geopolitical and economic uncertainty may therefore increase students' subsequent future development anxiety.

Conservation of resources theory proposes that when individuals perceive that important resources may be lost, they invest additional attentional and emotional resources in responding to the risk (Hobfoll, 1989). Students' sustained attention to future opportunities and developmental risks may reduce the psychological resources available for current academic tasks and weaken stable expectations regarding learning returns and future prospects. Prolonged resource depletion may manifest as learning exhaustion, detachment, and reduced academic efficacy (Salmela-Aro and Upadyaya, 2014; Schaufeli et al., 2002). Recent research has further shown that intolerance of uncertainty may be linked to academic burnout among university students through self-regulatory fatigue, providing empirical support for this resource-depletion process (Qiang et al., 2024). Perceived geopolitical and economic uncertainty may be concurrently associated with both future development anxiety and academic burnout at baseline. The extended model included these concurrent paths and the indirect associations arising from them and further tested the cross-temporal predictive effects of perceived geopolitical and economic uncertainty on T2 future development anxiety and T2 academic burnout. Related evidence has also linked future-oriented and employment-related anxiety to students' psychological adjustment (Kwak et al., 2025; Yang et al., 2025). The following hypotheses were therefore proposed:

**H1:** After controlling for T1 future development anxiety, T1 perceived geopolitical and economic uncertainty is positively associated with T2 future development anxiety.

**H2:** After controlling for T1 academic burnout, T1 perceived geopolitical and economic uncertainty is positively associated with T2 academic burnout.

### 2.2 Bidirectional relationship between future development anxiety and academic burnout

Future development anxiety may consume students' attentional and self-regulatory resources. When students repeatedly worry about employment, further study, and career development, they may find it difficult to maintain stable concentration and engagement in learning. A lack of confidence in future outcomes may also weaken their appraisal of the value of current study and increase learning exhaustion and detachment. Continued resource depletion may further reduce academic efficacy and increase the risk of academic burnout (Hobfoll, 1989; Tan et al., 2026).

Academic burnout may also increase future development anxiety. Greater learning exhaustion and detachment, together with lower academic efficacy, may lead students to evaluate their own abilities and future competitiveness less favorably. When students experience a prolonged lack of accomplishment in their studies, they may become more concerned that they will be unable to meet the requirements for further study or obtain desirable employment. Previous research has shown that academic burnout is closely associated with lower learning confidence, poorer psychological adjustment, and less favorable expectations for development (Salmela-Aro et al., 2009; Schaufeli et al., 2002; Wu et al., 2024). The following hypotheses were therefore proposed:

**H3a:** After controlling for T1 academic burnout, T1 future development anxiety positively predicts T2 academic burnout.
**H3b:** After controlling for T1 future development anxiety, T1 academic burnout positively predicts T2 future development anxiety.

### 2.3 Prospective-concurrent indirect association through future development anxiety

Students' appraisals of external change may coincide with concerns about employment, further study, and life stability. Such concerns can consume attention and weaken engagement in current academic tasks (Kong and Zeng, 2023; Tan et al., 2026). The present model therefore tested whether PGECU was indirectly associated with later academic burnout through FDA. Because parts of the tested paths were concurrent, this hypothesis concerns a prospective-concurrent indirect association rather than temporal mediation.

The focal analysis estimated the prospective-concurrent indirect association T1 PGECU → T2 FDA → T2 academic burnout (Cole and Maxwell, 2003; Maxwell and Cole, 2007). Extended analyses examined additional indirect associations involving concurrent T1 paths, autoregressive paths, and cross-wave paths.

**H4:** After adjustment for T1 future development anxiety and T1 academic burnout, T1 perceived geopolitical and economic uncertainty shows a prospective-concurrent indirect association with T2 academic burnout through T2 future development anxiety.

### 2.4 Moderating roles of employment competition pressure and perceived structural university support

Social cognitive career theory proposes that students' judgments about development opportunities, environmental barriers, and support resources influence their career adaptation and psychological state (Lent et al., 2000). When employment competition pressure is high, students are more likely to perceive that job opportunities are limited, employment requirements are increasing, or their own competitive advantages are insufficient. Previous research has found that university students' perceptions of declining employment opportunities are associated with greater employment pressure, whereas employment support and job-search self-efficacy can buffer this relationship (Yang et al., 2022). Geopolitical and economic uncertainty may then be more readily interpreted as a risk directly relevant to personal career development. Under these conditions, negative judgments about learning returns and future development may intensify, and the association between perceived geopolitical and economic uncertainty and academic burnout may become stronger. The following hypothesis was therefore proposed:

**H5:** Employment competition pressure positively moderates the relationship between T1 perceived geopolitical and economic uncertainty and T2 academic burnout.

The stress-buffering model of social support proposes that support resources can reduce the adverse psychological and behavioral effects of stress (Cohen and Wills, 1985). Academic guidance, employment services, psychological support, and development information provided by universities may increase students' perceived control over future development and help them adjust their academic and career plans. When perceived university support is high, students may obtain more information and coping resources even when employment competition is intense. Recent research also indicates that learning resources and social support help students cope with academic demands and are associated with lower academic burnout (Bakker and Mostert, 2024; Zhang et al., 2025). The strengthening effect of employment competition pressure on the relationship between external uncertainty and academic burnout may consequently be reduced. The following hypothesis was therefore proposed:

**H6:** Perceived structural university support negatively moderates the moderating effect proposed in H5.

## 3 Method

### 3.1 Research design and conceptual model

This study used a two-wave survey design. T1 was administered from November 10 to December 7, 2025, during the autumn recruitment season and end-of-term preparation. T2 was administered from May 9 to June 15, 2026, during spring recruitment and end-of-year examinations. T1 measured PGECU, FDA, academic burnout, ECP, PSUS, and demographic variables. T2 repeated FDA and academic-burnout measures. The interval was approximately five months.

Figure 1 presents the baseline-adjusted two-wave model. It included autoregressive and cross-lagged paths for FDA and academic burnout, T1 PGECU paths to both T2 outcomes, and the focal indirect association through T2 FDA. ECP and PSUS were included in two-way and three-way interactions. Concurrent T1 PGECU paths were estimated only as extended associations and were not interpreted temporally.

**Figure 1:**
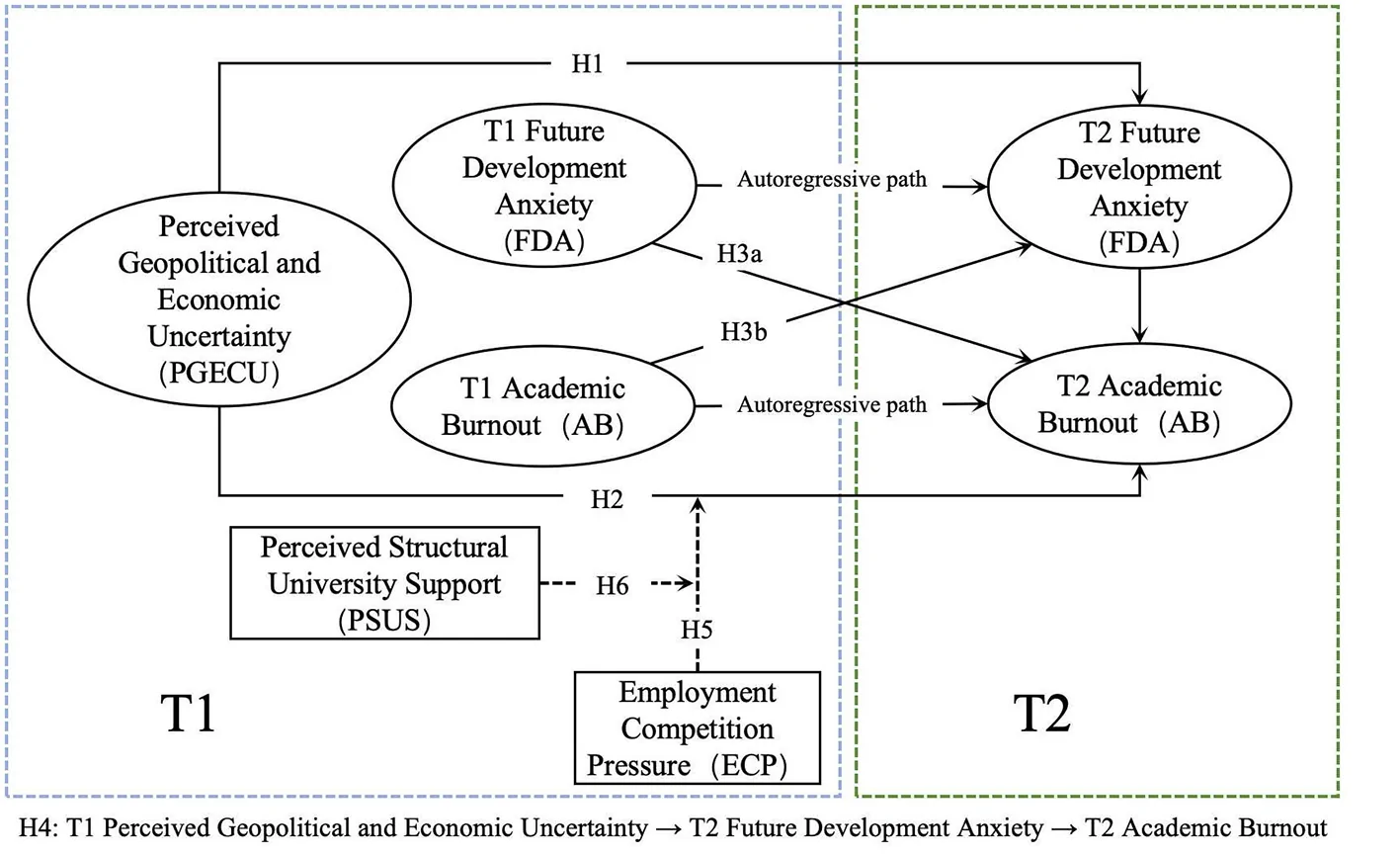
Conceptual model. T1 = baseline survey; T2 = follow-up survey. Solid arrows represent hypothesized structural associations, whereas dashed arrows represent hypothesized moderating effects.

### 3.2 Participants and procedure

Participants were undergraduate and postgraduate students from four universities in Zhengzhou, Henan Province, China. They were recruited through a nonprobability online convenience design and completed the survey through Wenjuanxing. Eligible participants were enrolled students who could understand the questionnaire, consent to two-wave matching, and generate an anonymous matching code. Records were excluded for failed wave matching, excessive core-item missingness, failed attention checks, implausibly short completion time, or mechanical response patterns. Institution identifiers, invitation denominators, channel-specific recruitment counts, incentives, and site-level response rates were not retained in the analytic files.

At T1, 1,823 participants provided analyzable baseline data. Of these, 1,215 provided valid matched two-wave data, while 608 did not contribute matched T2 data, corresponding to an attrition rate of 33.35%. The matched analytic sample had complete data for all modeled variables and required no imputation. The participant flow is shown in Figure 2.

**Figure 2:**
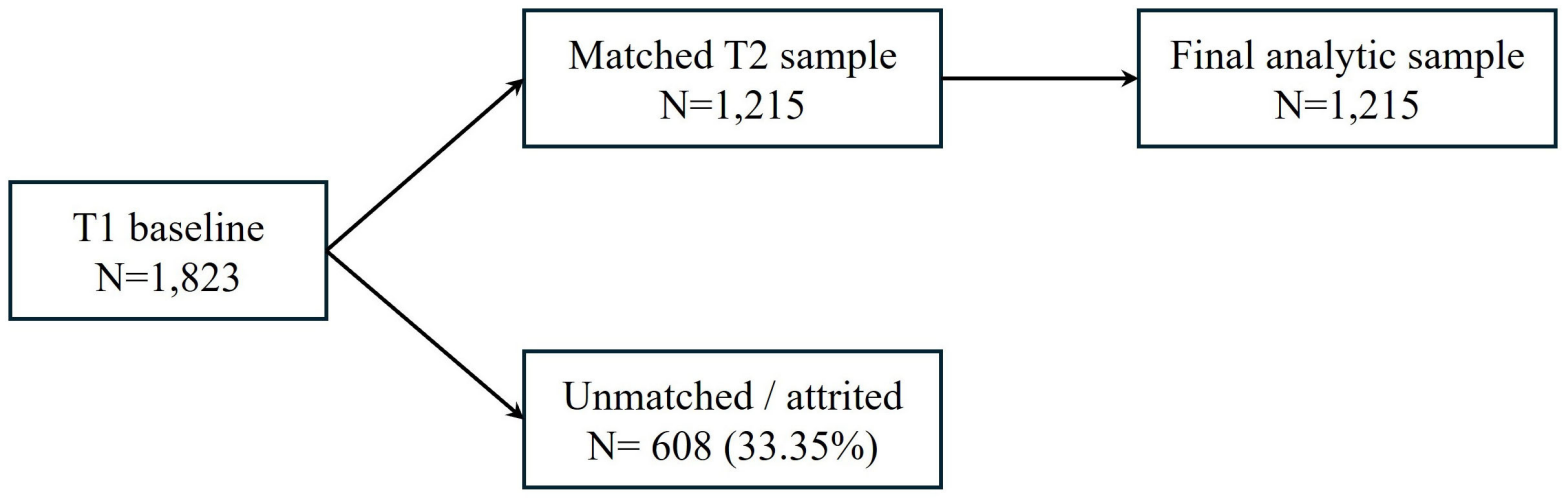
Participant flow diagram. T1 = baseline survey; T2 = follow-up survey. The final analytic sample comprised participants with valid T1–T2 matches and complete data for all modeled variables.

The study was reviewed and approved by the School Ethics Committee of the School of Marxism, North China University of Water Resources and Electric Power (Ref. NCWU/SRC/SOM/11; October 16, 2025). Electronic informed consent described the study, voluntary participation, withdrawal rights, data use, and privacy safeguards. Participants generated an eight-character code solely for matching T1 and T2 responses. The code contained no direct identifier, and no linkage to participants' identities was created or retained. Data were analyzed in de-identified form and reported in aggregate.

### 3.3 Measures

The five instruments were substantially adapted for this context; the cited source scales defined the construct domains but were not administered verbatim. PGECU, FDA, and ECP each comprised 6 items; academic burnout comprised 9 items; and PSUS comprised 8 items. All items used five-point Likert responses, and each construct score was the mean of its items. The final items were written in the direction of the named construct, so no reverse scoring was required. Supplementary Tables S1–S6 provide the complete questionnaire items.

Candidate items were developed after translating and reviewing construct definitions and representative source content. Representative source content included established measures of perceived stress, organizational support, career future orientation, and perceived social support (Cohen et al., 1983; Eisenberger et al., 1986; Rottinghaus et al., 2005; Zimet et al., 1988). Two bilingual researchers completed forward translation, a third completed back-translation, and five experts assessed relevance, clarity, cultural appropriateness, and dimensional coverage. I-CVI values ranged from 0.80 to 1.00, and S-CVI/Ave was 0.95. Cognitive interviews involved 12 students, and a separate pilot involved 180 students. 6 items were revised after piloting; none was deleted.

Academic burnout covered exhaustion, cynicism, and reduced efficacy. One-factor, correlated three-factor, and second-order specifications were compared. Near-unity dimension correlations and boundary estimates indicated that the dimensions were not empirically separable in this sample, so the nine-item mean was retained as a pragmatic composite. Alternative structures were also evaluated for PGECU and PSUS. Strong longitudinal measurement invariance was supported for FDA and academic burnout, but external validation remains necessary.

Control variables included gender, year, field, university type, postgraduation plan, family economic status, self-rated academic performance, international-news exposure, career-information exposure, and a recent major-event indicator. They were selected because demographic position, resources, academic standing, career plans, and information exposure may covary with the focal appraisals and outcomes. Categorical controls were dummy coded with explicit reference groups in Supplementary Table 4.31. Models with and without controls were compared because some controls may overlap conceptually with students' appraisals.

### 3.4 Data quality control and ethical considerations

Data were cleaned in a prespecified sequence. Records were excluded for failed attention checks, unmatched or nonunique codes, implausibly short completion times, mechanical response patterns, or more than 20% missingness on a core scale. The 1,215 matched cases had no missing values on analysis variables, so no imputation was used. Multivariate outliers were assessed with Mahalanobis distance based on the predictor set at each wave and a chi-square tail-probability threshold of *p* < 0.001. Final-model influence was also examined with Cook's distance and leverage.

Procedural safeguards included anonymous responding and temporal separation of the two waves. Harman's first-factor proportion was reported only as a preliminary diagnostic, not evidence that common-method bias was absent. A common-method-factor sensitivity model was also estimated. Because no marker variable was collected, residual common-method variance remains possible.

### 3.5 Data analysis strategy

Analyses proceeded from data screening to measurement-model and structural-model assessment. SPSS 27 was used for descriptive statistics and correlations. Attrition was examined across demographic, study-related, and five T1 core variables using group comparisons, effect sizes, a multivariable logistic model, and inverse-probability weighting. A sensitivity power analysis evaluated the smallest detectable two-way and three-way interaction effects (Baranger et al., 2023).

Wave-specific exploratory factor analyses used principal-axis extraction and Promax rotation (Kaiser, 1974). The sample was then randomly divided, using a fixed seed, into development (*n* = 607) and validation (*n* = 608) subsets. Covariance-based CFA used semopy 2.3.11 with maximum-likelihood estimation (Kline, 2023). Ordinal-sensitive analyses using polychoric correlations and DWLS were added as sensitivity checks. Competing factor structures, factor correlations, semantic-overlap sensitivity, and longitudinal measurement invariance were evaluated. Harman's test (Podsakoff et al., 2003) and a common-method-factor model were reported as limited diagnostics.

The structural model included autoregressive and cross-lagged paths, PGECU paths to both T2 outcomes, the T2 FDA-to-T2 burnout association, ECP and PSUS main effects, all constituent two-way interactions, the three-way interaction, and ten control-variable families. The classic two-wave cross-lagged specification estimates between-person prediction after baseline adjustment; it does not isolate within-person change. Observed-change-score models were therefore examined as a sensitivity analysis.

PLS-SEM (Hair and Alamer, 2022) was estimated in SmartPLS 4 using path weighting, a maximum of 300 iterations, and a 10∧-7 convergence criterion. Interactions used the two-stage procedure after mean-centering predictors. Inference used 5,000 bootstrap resamples, a fixed seed of 42, and bias-corrected 95% confidence intervals. PLS-SEM analyses used latent-variable estimates; OLS, Johnson-Neyman, and change-score analyses used scale means. Complete direct, indirect, interaction, control, and alternative-specification results are reported in the supplementary analysis files. The exact SmartPLS sign-change setting and statsmodels subversion should be verified before resubmission.

## 4 Results

### 4.1 Sample attrition and data screening

Attrition analyses used all 1,823 baseline participants, including 1,215 completers and 608 noncompleters. Demographic, study-related, and five T1 core variables showed no material group differences; all absolute standardized mean differences were at most 0.10. A 15-predictor logistic model did not significantly improve prediction of attrition (likelihood-ratio *p* = 0.847). Inverse-probability weighting changed the focal coefficient only from 0.152 to 0.154. At *N* = 1,215, the minimum detectable interaction effect was f-squared = 0.0065 at 80% power. Power exceeded 0.99 for the observed three-way interaction (f-squared = 0.024). Table 1, Supplementary Table 4.2 provide complete estimates and confidence intervals.

**Table 1:** Attrition analysis between participants who completed and did not complete T2.

Analysis	Statistic	Result
Demographic attrition comparisons	10 chi-square tests	All *p* > 0.05
T1 core-variable comparisons	*t* = −0.74 to 1.95	All *p* > 0.05; |*d*| < = 0.10
Multivariable attrition model	15 predictors	Likelihood-ratio *p* = 0.847
IPW sensitivity: PGECU -> T2 AB	beta 0.152 vs. 0.154	Both *p* < 0.001
Three-way interaction power	f-squared = 0.02	Power = 0.998

Retained at T2, *n* = 1,215; not retained at T2, *n* = 608. The *p* values were obtained from independent-samples *t* tests. PGECU, perceived geopolitical and economic uncertainty; FDA, future development anxiety; AB, academic burnout; ECP, employment competition pressure.

At T1, the maximum Mahalanobis distance was 13.120, below chi-square (4, 0.999) = 18.467. The earlier T2 calculation used one predictor and therefore reduced to a squared standardized distance. The analysis recalculated T2 distance using five final-model predictors; the maximum was 14.848, below chi-square (5, 0.999) = 20.515. Tail probabilities were obtained from the corresponding chi-square distribution. Removing the 10 cases with the largest Cook's distances changed core coefficients by 0.6%-10.9% without changing their directions.

### 4.2 Participant characteristics

The matched sample comprised 1,215 students. It included 1,140 undergraduates (93.8%) and 75 postgraduate students (6.2%); age was not collected. Gender was self-reported as male, female, or prefer not to say, with women comprising 51.9% and 29 participants selecting the third option. Third-year students were the largest group (26.6%). The most common fields were science and engineering (27.2%) and economics and management (20.1%). Participants attended comprehensive, science and engineering, normal, finance, medical, and other university types. Institution identifiers were unavailable, so site-level clustering could not be evaluated.

### 4.3 Descriptive statistics and correlations

Table 2 summarizes the study variables. PGECU correlated with T1 FDA (*r* = 0.390) and T1 academic burnout (*r* = 0.384). T1 FDA correlated with T1 academic burnout (*r* = 0.467), whereas PSUS correlated negatively with FDA and burnout (*r* = −0.161 to −0.222). Cross-wave correlations indicated moderate stability for FDA (*r* = 0.538) and academic burnout (*r* = 0.516). Using Cohen's approximate benchmarks, the principal correlations ranged from small-to-moderate to large. These are descriptive between-person associations and should not be interpreted causally.

**Table 2:** Descriptive statistics and correlations.

Variable	N	Mean (M)	SD	1	2	3	4	5	6	7
T1 PGECU	1215	3.024	1.008	1.000						
T1 FDA	1215	3.006	1.001	0.390^***^	1.000					
T1 AB	1215	3.007	0.969	0.384^***^	0.467^***^	1.000				
T1 ECP	1215	3.018	0.990	0.205^***^	0.327^***^	0.316^***^	1.000			
T1 PSUS	1215	2.998	0.974	−0.001	−0.161^***^	−0.187^***^	0.047	1.000		
T2 FDA	1215	3.015	0.981	0.411^***^	0.538^***^	0.342^***^	0.306^***^	−0.191^***^	1.000	
T2 AB	1215	3.011	0.932	0.416^***^	0.427^***^	0.516^***^	0.305^***^	−0.222^***^	0.441^***^	1.000

^*^p < 0.05. ^**^p < 0.01. ^***^p < 0.001. PGECU, perceived geopolitical and economic uncertainty; FDA, future development anxiety; AB, academic burnout; ECP, employment competition pressure; PSUS, perceived structural university support.

All 50 items were checked (Hatem et al., 2022) against the item-level export. Means ranged from 2.960 to 3.058, standard deviations from 1.188 to 1.290, skewness from approximately −0.07 to 0.07, and kurtosis from −1.075 to −0.884. Supplementary Table 4.5 reports counts and percentages for response categories 1-5 and the corresponding bar plots. Common five-point anchors, similarly directed wording, the large sample, and repeated measurement in one cohort may contribute to the regular distributions. Their regularity is reported descriptively and does not establish validity or eliminate the need for ordinal-sensitive analyses.

### 4.4 Exploratory factor analysis and common-method diagnostics

T1 and T2 were analyzed separately to avoid treating repeated measures as independent constructs. Principal-axis extraction with Promax rotation yielded the expected five-factor T1 solution and two-factor T2 solution. Primary loadings ranged from 0.682 to 0.789, and no cross-loading exceeded 0.40. The first unrotated factor explained 33.495% of variance, but this Harman result was treated only as preliminary. Adding an orthogonal method factor improved CFI by 0.007, indicating limited fit improvement. Neither diagnostic can exclude residual method variance, especially because no marker variable was collected.

### 4.5 Confirmatory factor analysis

Table 3 presents the covariance-based CFA results obtained using semopy 2.3.11 with maximum-likelihood estimation. The T1 five-factor model fit better than four-, three-, two-, and one-factor alternatives (chi-square = 1676.412, df = 550, CFI = 0.963, TLI = 0.960, RMSEA = 0.041). The T2 two-factor model also fit better than its one-factor alternative (chi-square = 447.83, df = 89, CFI = 0.981, TLI = 0.978, RMSEA = 0.058). Split-sample development and validation analyses produced similar loading patterns. Polychoric-correlation and DWLS sensitivity models led to the same substantive conclusion.

**Table 3:** Confirmatory factor analysis fit indices.

Model	chi-square	df	CFI	TLI	GFI	RMSEA
T1 five-factor	1676.412	550	0.963	0.960	0.925	0.041
T1 four-factor	3361.66	554	0.874	0.864	0.853	0.065
T1 three-factor	6062.37	557	0.752	0.735	0.734	0.090
T1 two-factor	10734.64	559	0.542	0.512	0.529	0.123
T1 one-factor	13279.09	560	0.427	0.392	0.418	0.137
T2 two-factor	447.83	89	0.981	0.978	0.966	0.058
T2 one-factor	3385.48	90	0.829	0.801	0.826	0.174

Academic-burnout scoring models, alternative PGECU and PSUS structures, and the complete latent-factor correlation matrix are reported in Supplementary Tables 4.14–4.16. Removing the three most semantically overlapping items changed factor correlations by at most 0.014 and core path coefficients by at most 4%. Configural, metric, and scalar invariance tests for FDA and academic burnout met the prespecified criteria of absolute change in CFI at most 0.010 and change in RMSEA at most 0.015 (Chen, 2007).

### 4.6 Model fit

CB-CFA was used to evaluate the measurement model, whereas SmartPLS 4 was used for prediction-oriented structural estimation and higher-order interactions. Reliability and convergent-validity indices met conventional criteria, and HTMT values were below 0.85. Indicator VIFs ranged from 2.029 to 5.987. Seven T2 burnout indicators exceeded the primary threshold of 5 but remained below 6. This local collinearity is consistent with the semantic overlap among several burnout items and the near-unity correlations among the burnout dimensions observed in this sample. Removing these indicators changed coefficient magnitude but not direction or significance. All 9 items were therefore retained to preserve coverage of exhaustion, cynicism, and reduced efficacy. Exact item-level VIF values are reported in the Supplementary material.

The extended model explained 30.4% of T2 FDA variance and 45.5% of T2 academic-burnout variance. SRMR was 0.028 and NFI was 0.929. Local f-squared values were 0.137 for T1-to-T2 FDA stability, 0.071 for T1-to-T2 burnout stability, and 0.024 for the three-way interaction. The interaction should therefore be interpreted as small, despite its statistical precision. CB-SEM and observed-score sensitivity models were used to assess estimator dependence.

### 4.7 Path analysis

H1–H6 were treated as theory-specified tests. Johnson-Neyman regions, (Carden et al., 2017) the significance heatmap, influence diagnostics, observed-change scores, and control specifications were exploratory robustness analyses. The study was not preregistered, so model-selection and Type I error risks remain. A Holm correction across structural paths was added as a sensitivity analysis; the core hypothesis decisions were unchanged.

Figure 3 presents the estimated results of the extended structural model. After baseline adjustment, T1 PGECU was positively associated with T2 FDA (beta = 0.175, 95% CI [0.119, 0.232]) and T2 academic burnout (beta = 0.148, 95% CI [0.091, 0.206]). T1 FDA predicted T2 academic burnout (beta = 0.176, 95% CI [0.117, 0.238]), and T1 burnout predicted T2 FDA (beta = 0.087, 95% CI [0.029, 0.143]). Autoregressive paths were also significant for FDA (beta = 0.387, 95% CI [0.331, 0.438]) and burnout (beta = 0.250, 95% CI [0.196, 0.307]). These estimates represent cross-wave between-person prediction. They do not show that within-person change in one construct caused later within-person change in another.

**Figure 3:**
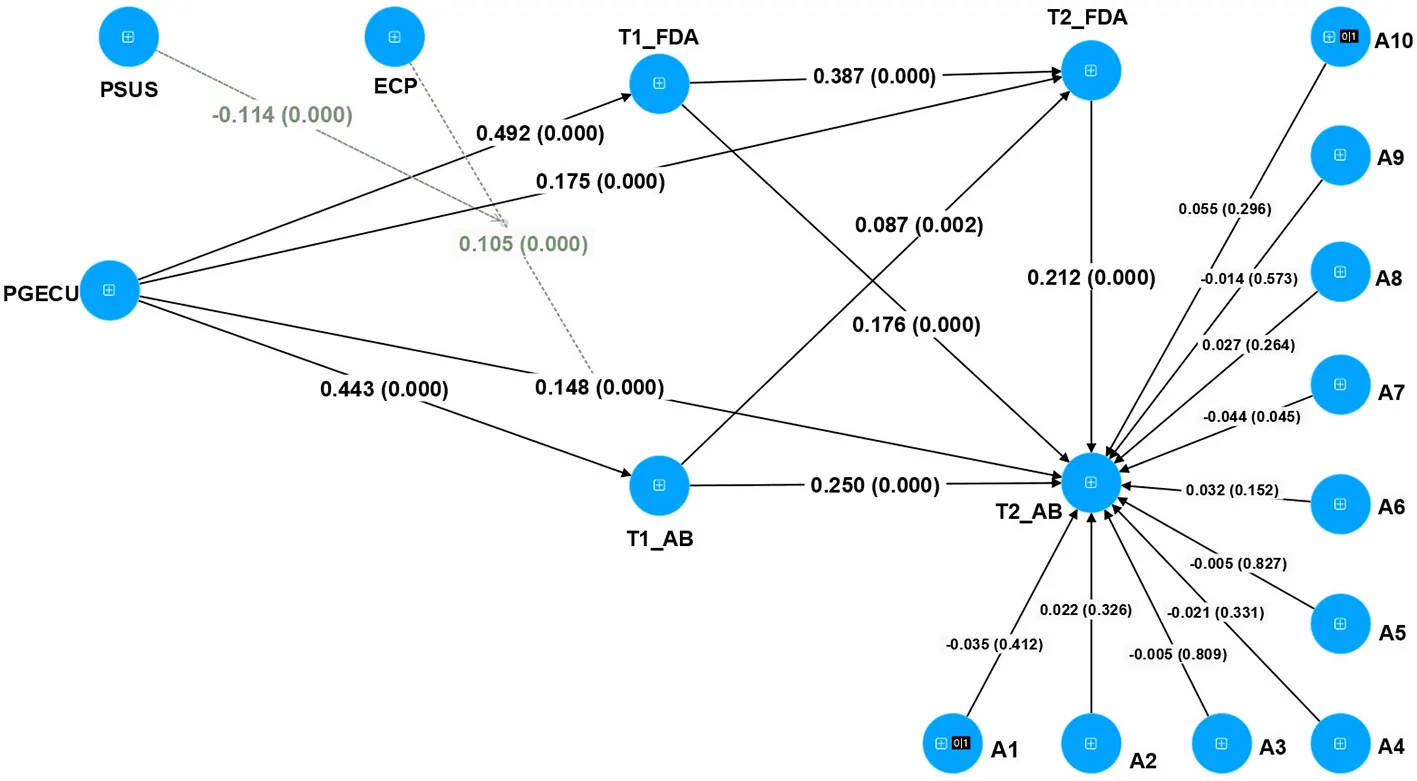
Estimated results of the extended structural model. Path values are standardized coefficients, and values in parentheses are *p* values obtained from 5,000 bootstrap resamples. Values displayed as 0.000 indicate *p* < 0.001. A1–A10 denote the ten control-variable families. T1 = baseline survey; T2 = follow-up survey. PGECU, perceived geopolitical and economic uncertainty; FDA, future development anxiety; AB, academic burnout; ECP, employment competition pressure; PSUS, perceived structural university support.

The focal prospective-concurrent indirect association T1 PGECU → T2 FDA → T2 academic burnout was significant (beta = 0.037, 95% CI [0.024, 0.054]). Its second component was the concurrent T2 FDA -> T2 burnout path (beta = 0.212, 95% CI [0.157, 0.267]). Additional indirect associations through T1 FDA, T1 burnout, and autoregressive paths were also significant. Because each indirect path contained at least one concurrent segment, these estimates combine concurrent association with cross-wave prediction and do not establish temporal mediation.

The ECP × PGECU interaction was positive (beta = 0.105, 95% CI [0.065, 0.142]), indicating a stronger PGECU-burnout association at higher ECP. The PSUS × ECP × PGECU interaction was negative (beta = −0.114, 95% CI [−0.152, −0.078]), indicating that this moderation weakened as PSUS increased. The ECP × PSUS and PGECU × PSUS two-way interactions were not significant. Table 4 reports the complete focal coefficients and confidence intervals.

**Table 4:** Path analysis.

Path	beta	SE	*p*	95% CI (BCa)
Direct paths				
T1 AB -> T2 AB	0.250	0.028	< 0.001	[0.196, 0.307]
T1 FDA -> T2 FDA	0.387	0.027	< 0.001	[0.331, 0.438]
PGECU -> T2 AB	0.148	0.029	< 0.001	[0.091, 0.206]
PGECU -> T2 FDA	0.175	0.029	< 0.001	[0.119, 0.232]
T1 FDA -> T2 AB	0.176	0.031	< 0.001	[0.117, 0.238]
T1 AB -> T2 FDA	0.087	0.029	0.002	[0.029, 0.143]
T2 FDA -> T2 AB	0.212	0.028	< 0.001	[0.157, 0.267]
ECP -> T2 AB	0.053	0.025	0.038	[0.002, 0.101]
PSUS -> T2 AB	−0.059	0.024	0.016	[−0.106, −0.009]
Indirect associations				
PGECU -> T1 FDA -> T2 FDA -> T2 AB	0.040	0.006	< 0.001	[0.029, 0.054]
PGECU -> T2 FDA -> T2 AB	0.037	0.008	< 0.001	[0.024, 0.054]
PGECU -> T1 FDA -> T2 AB	0.086	0.016	< 0.001	[0.057, 0.119]
PGECU -> T1 AB -> T2 AB	0.111	0.014	< 0.001	[0.085, 0.138]
PGECU -> T1 AB -> T2 FDA -> T2 AB	0.008	0.003	0.006	[0.003, 0.015]
Interaction paths				
ECP x PGECU -> T2 AB	0.105	0.020	< 0.001	[0.065, 0.142]
PSUS x ECP x PGECU -> T2 AB	−0.114	0.019	< 0.001	[−0.152,−0.078]
PSUS x ECP -> T2 AB	0.015	0.023	0.511	[−0.029, 0.059]
PSUS x PGECU -> T2 AB	0.029	0.021	0.164	[−0.012, 0.068]

beta = standardized coefficient; SE = bootstrap standard error; CI = bias-corrected 95% bootstrap confidence interval based on 5,000 resamples. Complete structural, control, alternative-specification, and Holm-adjusted results are reported in Supplementary Table 4.20.

PGECU, perceived geopolitical and economic uncertainty; FDA, future development anxiety; AB, academic burnout; ECP, employment competition pressure; PSUS, perceived structural university support.

As shown in Figure 4, mean-centered simple-slope analyses showed a stronger PGECU-burnout association at higher ECP. The conditional slopes were 0.095 at one standard deviation below the ECP mean, 0.191 at the mean, and 0.287 at one standard deviation above the mean; all were estimated with 95% confidence intervals. The joint pattern across ECP and PSUS was consistent with the negative three-way coefficient (Figure 5).

**Figure 4:**
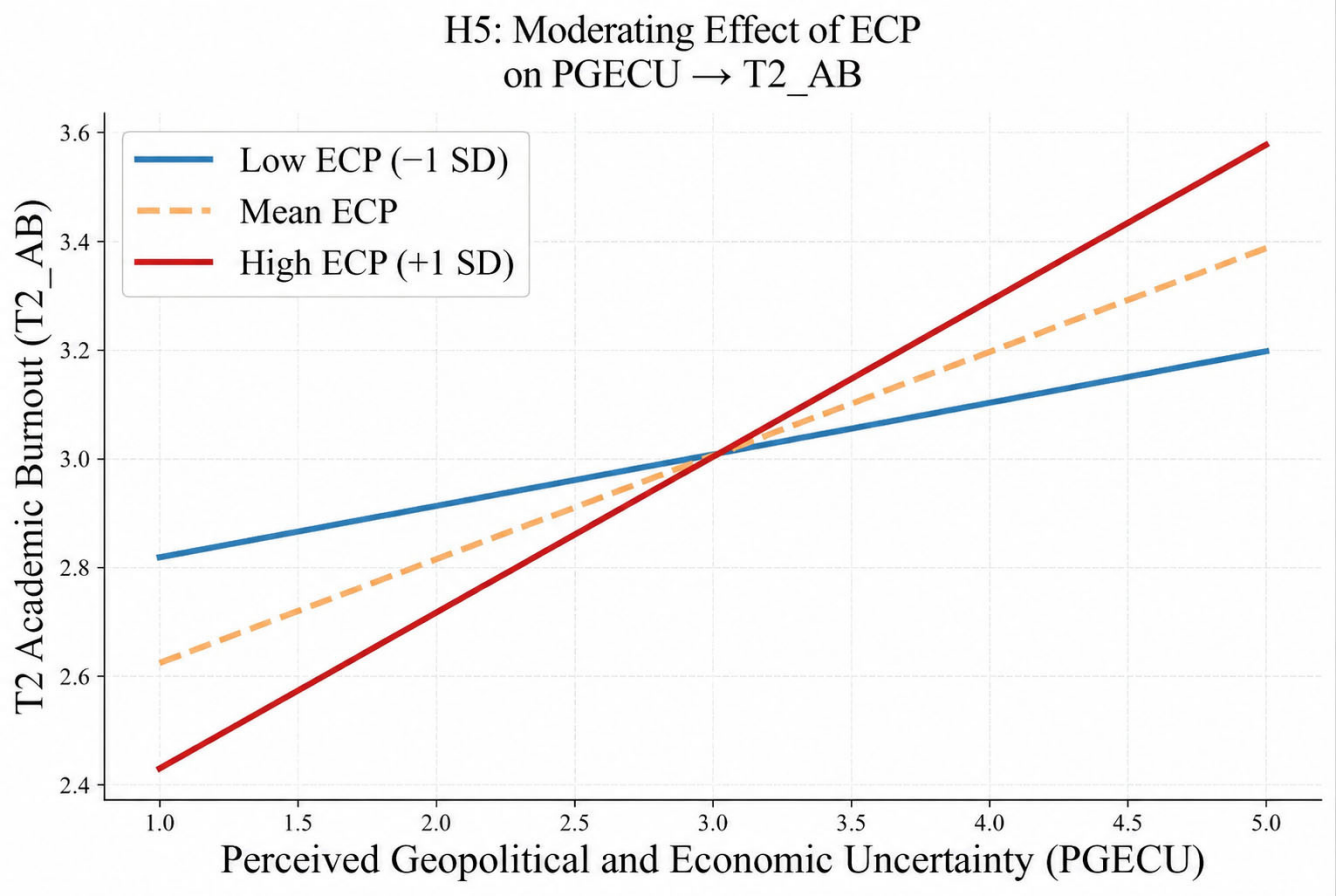
Moderating effect of employment competition pressure. The figure presents estimated simple slopes for the association between T1 perceived geopolitical and economic uncertainty and T2 academic burnout at low (−1 SD), mean, and high (+1 SD) levels of employment competition pressure.

**Figure 5:**
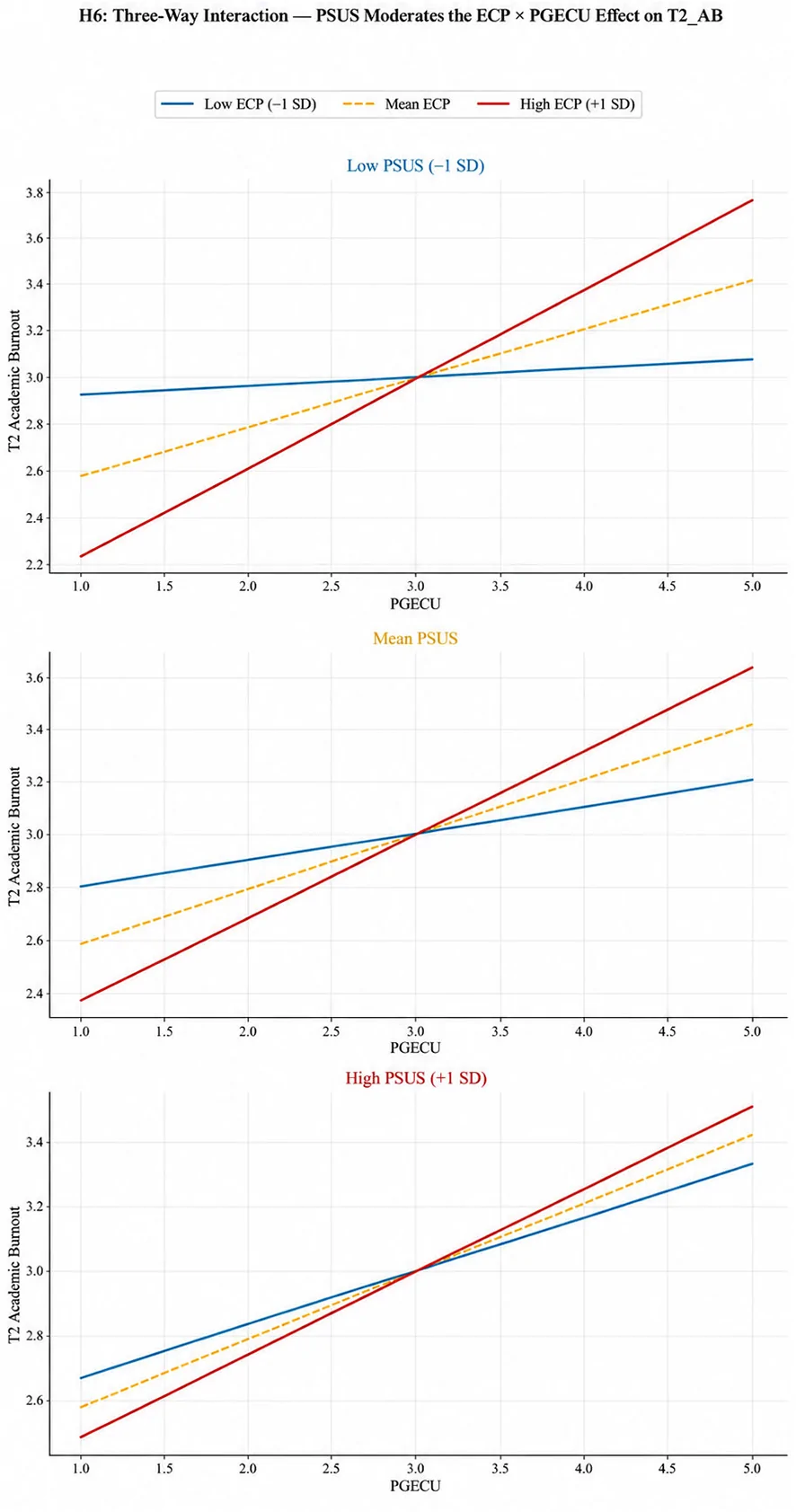
Joint moderating effects of perceived structural university support and employment competition pressure. The figure presents the three-way interaction among T1 perceived geopolitical and economic uncertainty, employment competition pressure, and perceived structural university support in relation to T2 academic burnout. The panels represent low, mean, and high levels of perceived structural university support; the lines represent low, mean, and high levels of employment competition pressure.

Johnson-Neyman analyses located the ECP threshold at 1.76 on the 1–5 scale, near the lower observed range (Figure 6). The PGECU-burnout association was significant above this value. The ECP × PGECU interaction was significant when PSUS was below 3.89, a value above the scale midpoint, and nonsignificant at higher PSUS. Supplementary Tables 4.28–4.29 provide the complete conditional-effect grid, confidence intervals, VIFs, and reproducible Johnson-Neyman output.

**Figure 6:**
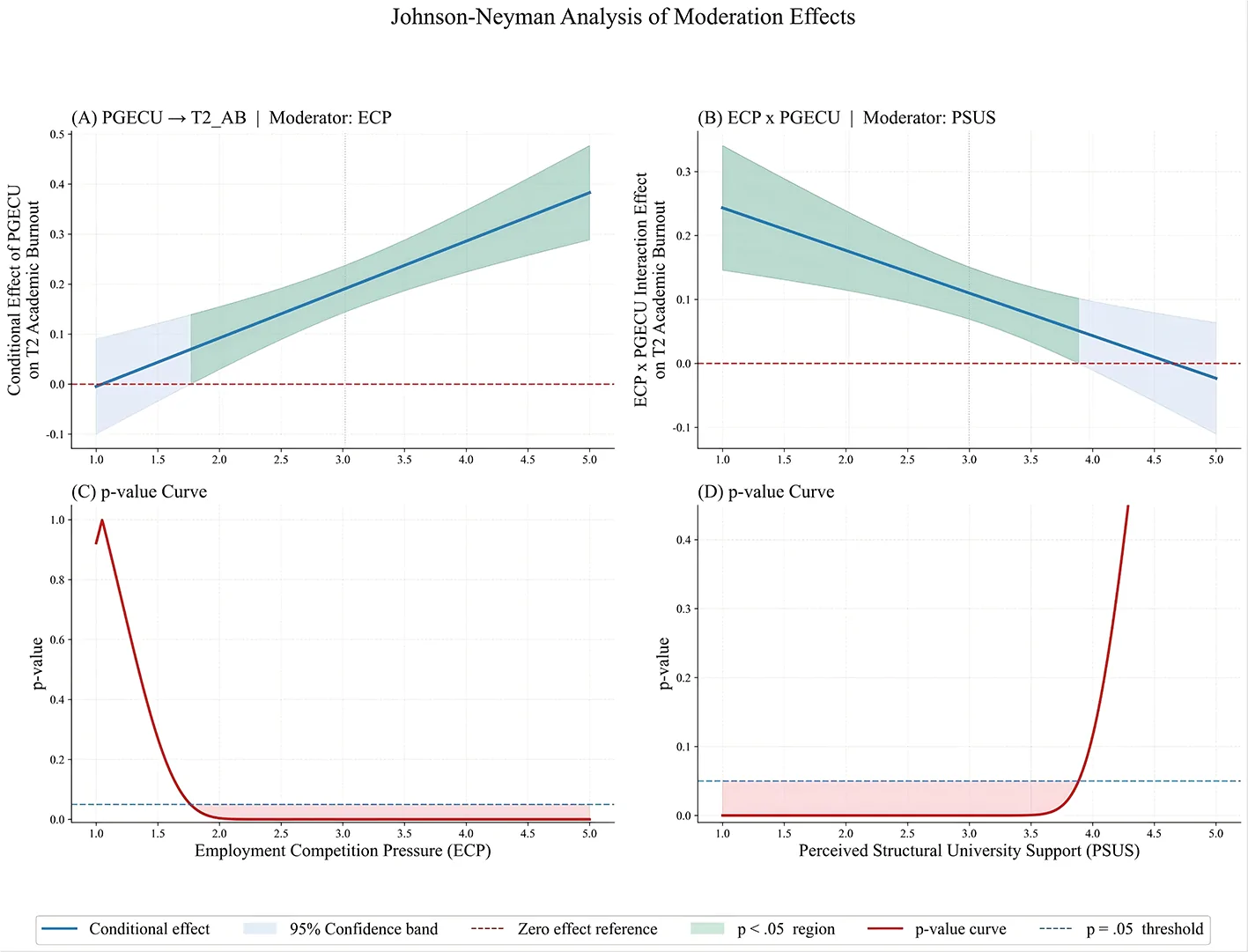
Johnson–Neyman Plots of the Moderation Effects. Panel **(A)** shows the conditional association between T1 perceived geopolitical and economic uncertainty and T2 academic burnout across levels of employment competition pressure. Panel **(B)** shows the conditional effect of the interaction between employment competition pressure and T1 perceived geopolitical and economic uncertainty on T2 academic burnout across levels of perceived structural university support. Panels **(C)** and **(D)** show the corresponding *p*-value curves; shaded regions indicate *p* < 0.05.

Models with and without the ten control-variable families produced similar core coefficients; R-squared changed from 0.414 to 0.419. Complete control coefficients, coding rules, reference groups, nonsignificant paths, and alternative specifications are reported in Supplementary Tables 4.20, 4.30–4.31.

The significance heatmap in Figure 7 further shows that the conditional effect of perceived geopolitical and economic uncertainty was strongest under low perceived structural university support and high employment competition pressure and weakened as perceived structural university support increased.

**Figure 7:**
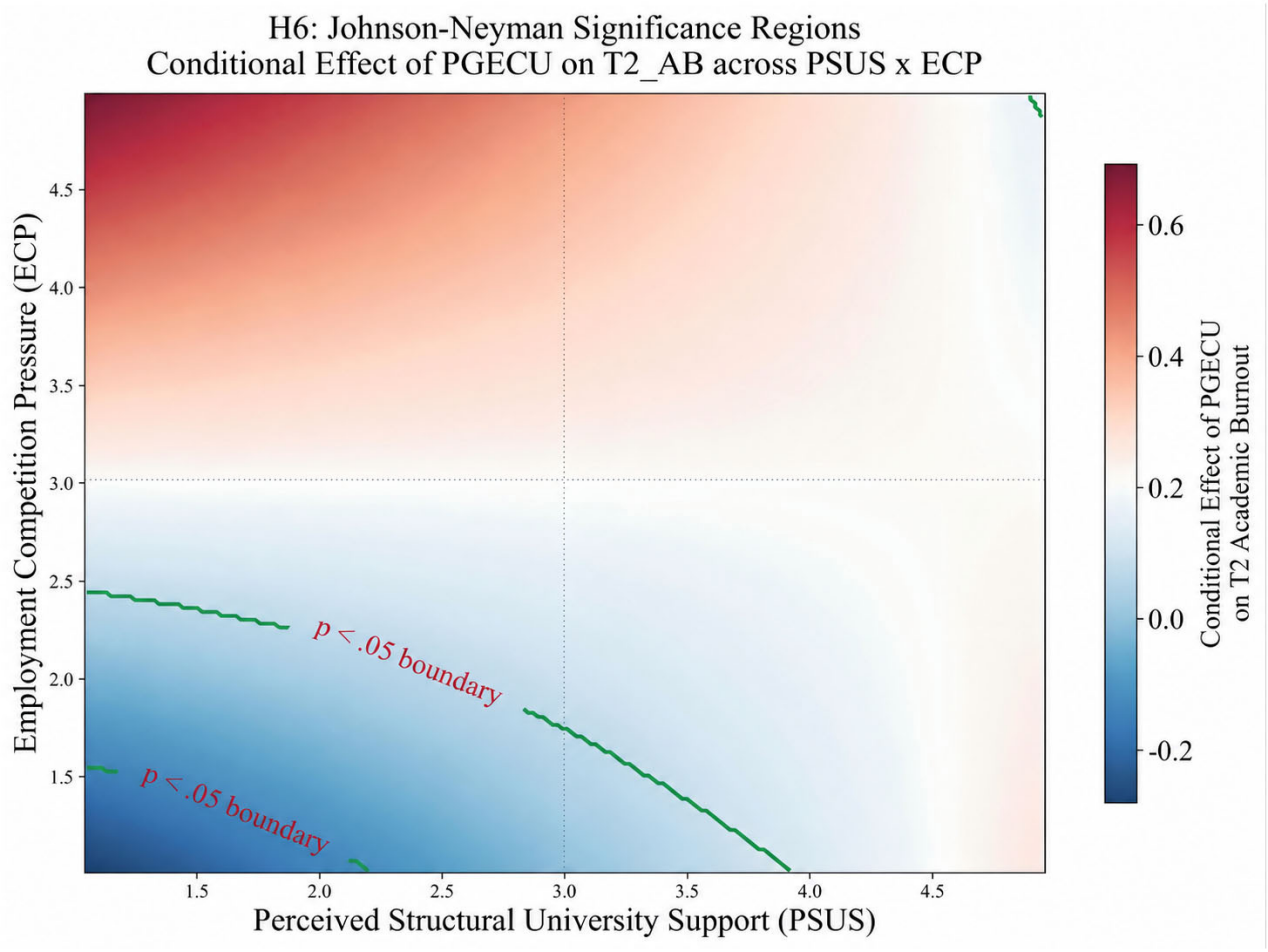
Full Johnson–Neyman region-of-significance heatmap. Colors represent estimated conditional associations: warmer colors indicate stronger positive associations, whereas cooler colors indicate weaker or negative associations. Green contours mark the *p* =0.05 boundary, and dotted lines indicate the scale midpoint. PGECU, perceived geopolitical and economic uncertainty; PSUS, perceived structural university support; ECP, employment competition pressure; AB, academic burnout.

## 5 Discussion

### 5.1 Main findings

This study examined whether PGECU showed cross-wave associations with FDA and academic burnout. T1 PGECU predicted both T2 outcomes after baseline adjustment, but the effect sizes were small to moderate. This pattern is consistent with cognitive appraisal theory because students' interpretations of external change, rather than objective events alone, were associated with later academic functioning. However, stable individual differences, unmeasured time-varying factors, and measurement error remain alternative explanations.

The focal prospective-concurrent indirect association through T2 FDA was statistically reliable. This pattern is consistent with the proposition that uncertainty appraisals and future-oriented worry are linked to academic functioning. It does not establish a psychological mechanism because T2 FDA and burnout were measured concurrently. A three-wave design is required to test whether earlier PGECU precedes later FDA, which in turn precedes subsequent burnout.

FDA and academic burnout also showed asymmetric cross-wave prediction. Higher T1 FDA was associated with higher T2 burnout, and the reverse association was weaker. These coefficients describe differences between students after baseline adjustment; they do not show that changing one student's FDA changes that student's later burnout. The pattern is compatible with resource-depletion and self-efficacy accounts, but random-intercept models with at least three waves are needed to separate stable traits from within-person dynamics.

The PGECU-burnout association was stronger at higher ECP. This interaction was statistically precise but small in local effect size. ECP may make external uncertainty more personally relevant to employment and career planning, although the nonexperimental design cannot establish that ECP amplifies risk within individuals.

The three-way interaction indicated that the ECP moderation weakened as PSUS increased. This pattern is consistent with stress-buffering theory and social cognitive career theory because accessible guidance and support may alter students' appraisals of career barriers. The result does not demonstrate that university support prevents burnout. Intervention studies are needed to test whether changing services improves academic or psychological outcomes.

### 5.2 Theoretical implications

The theoretical contributions of this study are reflected in three areas.

First, this study incorporated perceived geopolitical and economic uncertainty into research on academic burnout among university students, thereby extending the explanatory boundaries of academic burnout. Previous research has generally explained academic burnout in terms of course workload, academic pressure, school adjustment, and individual psychological characteristics. Although this approach is necessary, it may confine academic burnout to the internal campus context. The present findings indicate that academic burnout among university students may also be associated with the broader socioeconomic environment. More precisely, it is not international events themselves but students' subjective judgments about the relationship of those events to their future development that are relevant to academic burnout. This finding links macro-level uncertainty, individual risk appraisal, and academic functioning and provides a more complete framework for understanding academic burnout among contemporary university students.

Second, the study identified a prospective-concurrent indirect association involving FDA. Cognitive appraisal theory specifies why macro-level uncertainty may become personally salient, whereas conservation of resources theory explains why sustained worry may coincide with reduced academic functioning. The concurrent T2 segment prevents a temporal mechanism claim, so the result is best treated as a hypothesis for future three-wave research.

Third, ECP and PSUS identified complementary boundary conditions. Social cognitive career theory situates career barriers and institutional resources within students' developmental appraisals. Stress-buffering theory motivates the prediction that support attenuates a stress-related association. Together with cognitive appraisal and resource-conservation accounts, these theories address distinct stages of the conceptual model rather than serving as interchangeable explanations.

### 5.3 Practical implications

The findings may help universities identify topics for student-support assessment, particularly future-oriented worry and perceived employment competition. Any screening should be voluntary, confidential, and linked to accessible support. The present estimates do not show that screening prevents burnout, so implementation should be evaluated rather than assumed effective.

Career services may consider providing clearer labor-market information, planning support, and referral pathways for students reporting high ECP. Because the interaction effect was small, these services should complement broader academic and mental-health support rather than target students solely on the basis of a questionnaire score.

Coordinated academic advising, career services, and counseling may increase the visibility and accessibility of university support. The three-way interaction suggests a potential buffering pattern, but it does not establish that service redesign will reduce burnout. Controlled implementation studies should test reach, uptake, and student outcomes.

### 5.4 Limitations and future research

This study has several limitations. First, T2 FDA and academic burnout were concurrent, so the indirect estimates do not establish temporal mediation. Second, the classic two-wave cross-lagged model mixes stable between-person differences with within-person change; at least three waves are needed for a random-intercept model. Third, all focal constructs were self-reported. Harman and method-factor diagnostics cannot eliminate residual common-method bias. Fourth, the context-specific measures require independent external validation. Several items overlap semantically, and the composite burnout score is a pragmatic scoring choice rather than proof of strict unidimensionality. Fifth, the online convenience sample came from four universities in one city. Age, site identifiers, invitation denominators, site-specific response rates, and recruitment-channel counts were unavailable, and institutional clustering could not be modeled. Sixth, the study was not preregistered and combined planned hypotheses with exploratory robustness analyses. Holm adjustment reduced but did not eliminate model-selection and multiplicity concerns. Finally, most structural and interaction effects were small. Practical recommendations should therefore be tested in intervention studies before being treated as preventive strategies.

## 6 Conclusion

In this two-wave sample, PGECU showed small to moderate cross-wave associations with FDA and academic burnout. FDA and burnout showed asymmetric cross-wave associations, and PGECU showed a prospective-concurrent indirect association with T2 burnout through T2 FDA. ECP was associated with a stronger focal relationship, whereas this moderation weakened at higher PSUS. These findings define testable longitudinal and intervention hypotheses but do not establish within-person causation, temporal mediation, or preventive effects of university support.

## Data Availability

The raw data supporting the conclusions of this article will be made available by the authors, without undue reservation.

## References

[B1] Bakker A. B. Mostert K. (2024). Study demands–resources theory: understanding student wellbeing in higher education. Educ. Psychol. Rev. 36:92. doi: 10.1007/s10648-024-09940-8

[B2] Baranger D. A. Finsaas M. C. Goldstein B. L. Vize C. E. Lynam D. R. Olino T. M. (2023). Tutorial: power analyses for interaction effects in cross-sectional regressions. Adv. Methods Pract. Psychol. Sci. 6:25152459231187531. doi: 10.1177/25152459231187531 PMC12341451 40799847

[B3] Carden S. W. Holtzman N. S. Strube M. J. (2017). CAHOST: an excel workbook for facilitating the Johnson-Neyman technique for two-way interactions in multiple regression. Front. Psychol. 8:1293. doi: 10.3389/fpsyg.2017.01293 PMC5532434 28804471

[B4] Carleton R. N. Norton M. P. J. Asmundson G. J. (2007). Fearing the unknown: a short version of the Intolerance of Uncertainty Scale. J. Anxiety Disord. 21, 105–117. doi: 10.1016/j.janxdis.2006.03.014 16647833

[B5] Chen F. F. (2007). Sensitivity of goodness of fit indexes to lack of measurement invariance. Struct. Equ. Modeling 14, 464–504. doi: 10.1080/10705510701301834

[B6] Cohen S. Kamarck T. Mermelstein R. (1983). A global measure of perceived stress. J. Health Soc. Behav. 24, 385–396. doi: 10.2307/2136404 6668417

[B7] Cohen S. Wills T. A. (1985). Stress, social support, and the buffering hypothesis. Psychol. Bull. 98:310. doi: 10.1037/0033-2909.98.2.310 3901065

[B8] Cole D. A. Maxwell S. E. (2003). Testing mediational models with longitudinal data: questions and tips in the use of structural equation modeling. J. Abnorm. Psychol. 112:558. doi: 10.1037/0021-843X.112.4.558 14674869

[B9] Eisenberger R. Huntington R. Hutchison S. Sowa D. (1986). Perceived organizational support. J. Appl. Psychol. 71:500. doi: 10.1037/0021-9010.71.3.500

[B10] Freeston M. H. Rhéaume J. Letarte H. Dugas M. J. Ladouceur R. (1994). Why do people worry? Pers. Individ. Dif. 17, 791–802. doi: 10.1016/0191-8869(94)90048-5

[B11] Grupe D. W. Nitschke J. B. (2013). Uncertainty and anticipation in anxiety: an integrated neurobiological and psychological perspective. Nat. Rev. Neurosci. 14, 488–501. doi: 10.1038/nrn3524 PMC4276319 23783199

[B12] Hair J. Alamer A. (2022). Partial least squares structural equation modeling (PLS-SEM) in second language and education research: guidelines using an applied example. Res. Methods Appl. Linguist. 1:100027. doi: 10.1016/j.rmal.2022.100027

[B13] Hatem G. Zeidan J. Goossens M. Moreira C. (2022). Normality testing methods and the importance of skewness and kurtosis in statistical analysis. BAU J.-Sci. Technol. 3:7. doi: 10.54729/KTPE9512

[B14] Hobfoll S. E. (1989). Conservation of resources: a new attempt at conceptualizing stress. Am. Psychol. 44:513. doi: 10.1037/0003-066X.44.3.513 2648906

[B15] Kaiser H. F. (1974). An index of factorial simplicity. Psychometrika 39, 31–36. doi: 10.1007/BF02291575

[B16] Kline R. B. (2023). Principles and Practice of Structural Equation Modeling. New York, NY: Guilford publications.

[B17] Kong T. Zeng S. (2023). The effect of perceived environmental uncertainty on university students' anxiety, academic engagement, and prosocial behavior. Behav. Sci. 13:906. doi: 10.3390/bs13110906 PMC10669797 37998653

[B18] Kwak Y. Kim Y. Chae H. (2025). Job search anxiety and flourishing among university students: the serial mediating effects of social support and strengths use. BMC Psychol. 13, 652. doi: 10.1186/s40359-025-02995-4 PMC12211445 40597452

[B19] Lazarus R. S. Folkman S. (1984). Stress, Appraisal, and Coping. New York, NY: Springer publishing company.

[B20] Lent R. W. Brown S. D. Hackett G. (2000). Contextual supports and barriers to career choice: a social cognitive analysis. J. Couns. Psychol. 47:36. doi: 10.1037/0022-0167.47.1.36

[B21] Liu Z. Xie Y. Sun Z. Liu D. Yin H. Shi L. (2023). Factors associated with academic burnout and its prevalence among university students: a cross-sectional study. BMC Med. Educ. 23:317. doi: 10.1186/s12909-023-04316-y PMC10163855 37149602

[B22] Maxwell S. E. Cole D. A. (2007). Bias in cross-sectional analyses of longitudinal mediation. Psychol. Methods 12:23. doi: 10.1037/1082-989X.12.1.23 17402810

[B23] Podsakoff P. M. MacKenzie S. B. Lee J.-Y. Podsakoff N. P. (2003). Common method biases in behavioral research: a critical review of the literature and recommended remedies. J. Appl. Psychol. 88:879. doi: 10.1037/0021-9010.88.5.879 14516251

[B24] Qiang J. He X. Xia Z. Huang J. Xu C. (2024). The association between intolerance of uncertainty and academic burnout among university students: the role of self-regulatory fatigue and self-compassion. Front Public Health 12:1441465. doi: 10.3389/fpubh.2024.1441465 39114523 PMC11303341

[B25] Rottinghaus P. J. Day S. X. Borgen F. H. (2005). The career futures inventory: a measure of career-related adaptability and optimism. J. Career. Assess. 13, 3–24. doi: 10.1177/1069072704270271

[B26] Salmela-Aro K. Kiuru N. Leskinen E. Nurmi J.-E. (2009). School burnout inventory (SBI) reliability and validity. Eur. J. Psychol. Assess. 25, 48–57. doi: 10.1027/1015-5759.25.1.48

[B27] Salmela-Aro K. Upadyaya K. (2014). School burnout and engagement in the context of demands–resources model. BritishJ. Educ. Psychol. 84, 137–151. doi: 10.1111/bjep.12018 24547758

[B28] Schaufeli W. B. Martinez I. M. Pinto A. M. Salanova M. Bakker A. B. (2002). Burnout and engagement in university students: a cross-national study. J. Cross Cult. Psychol. 33, 464–481. doi: 10.1177/0022022102033005003

[B29] Tan Y. Liu S. Zang L. (2026). Future anxiety and academic burnout among university students: a short-term three-wave longitudinal conditional process study. BMC Psychol. 14:1128. doi: 10.1186/s40359-026-04933-4 PMC13435513 42237196

[B30] Wu X. Zhang W. Li Y. Zheng L. Liu J. Jiang Y. Peng Y. (2024). The influence of big five personality traits on anxiety: the chain mediating effect of general self-efficacy and academic burnout. PLOS ONE 19:e0295118. doi: 10.1371/journal.pone.0295118 PMC10760915 38166098

[B31] Yang M. Li X. Qin X. Tian X. Zhang H. Wen H. (2025). The relationship between perceived academic stress and college students' employment anxiety: the mediating role of psychological resilience. Front. Psychiat. 16:1602808. doi: 10.3389/fpsyt.2025.1602808 PMC12170629 40530071

[B32] Yang S. Yang J. Yue L. Xu J. Liu X. Li W. Cheng H. He G. (2022). Impact of perception reduction of employment opportunities on employment pressure of college students under COVID-19 epidemic–joint moderating effects of employment policy support and job-searching self-efficacy. Front. Psychol. 13:986070. doi: 10.3389/fpsyg.2022.986070 PMC9627308 36337528

[B33] Zaleski Z. (1996). Future anxiety: concept, measurement, and preliminary research. Pers. Individ. Dif. 21, 165–174. doi: 10.1016/0191-8869(96)00070-0

[B34] Zhang J. Meng J. Wen X. (2025). The relationship between stress and academic burnout in college students: evidence from longitudinal data on indirect effects. Front. Psychol. 16:1517920. doi: 10.3389/fpsyg.2025.1517920 40491945 PMC12146318

[B35] Zimet G. D. Dahlem N. W. Zimet S. G. Farley G. K. (1988). The multidimensional scale of perceived social support. J. Pers. Assess. 52, 30–41. doi: 10.1207/s15327752jpa5201_2 2280326

