# Supplementary material for: Academic burnout under geopolitical and economic uncertainty: longitudinal associations with future development anxiety, employment competition pressure, and perceived structural university support
Source: Front Psychol. 2026 Sep 28;17:1951855. doi: 10.3389/fpsyg.2026.1951855 (PMC13654946; doi:10.3389/fpsyg.2026.1951855)
Supplement: Supplementary file 1 [file Supplementary_file_1.docx]

Academic Burnout Under Geopolitical and Economic Uncertainty: Longitudinal Associations With Future Development Anxiety, Employment Competition Pressure, and Perceived Structural University Support

^1^**Qingzhe Zhang**

^1^ The school of Marxism, North China University of Water Resources and Electric Power, Zhengzhou 450046, China.

*** Correspondence:**

Qingzhe Zhang

[zhangqingzhe3111@126.com](mailto:zhangqingzhe3111@126.com)

**Keywords: academic burnout; future development anxiety; geopolitical and economic uncertainty; employment competition pressure; perceived structural university support**

**Appendix 1**

**1 Measurement Instruments**

The construct definitions and candidate items of the English-language source scales were independently translated by two bilingual Chinese-English researchers. One researcher had expertise in educational psychology and psychometrics, and the other had expertise in applied linguistics and academic translation. After the translations were reconciled, a preliminary Chinese version was produced and back-translated by a bilingual researcher who had not participated in the forward translation. Five experts in educational psychology, higher education, psychometrics, university student career development, and language studies then reviewed the relevance, clarity, cultural appropriateness, and dimensional representativeness of the items. Based on their feedback, the research team revised eight items. Item-level content validity index (I-CVI) values ranged from 0.80 to 1.00, and the scale-level content validity index based on the average method (S-CVI/Ave) was 0.95. Before the formal survey, cognitive interviews were conducted with 12 university students from different years and fields of study, and a pilot test was administered to 180 university students who were not included in the formal sample. The pilot assessment focused on item comprehension, response burden, corrected item-total correlations, internal consistency, and the preliminary factor structure. Based on the results, six items were revised and no items were deleted, leaving a final set of 35 items. Cronbach's α values for the five scales were 0.89, 0.91, 0.93, 0.88, and 0.94, respectively, and corrected item-total correlations ranged from 0.53 to 0.81.

**Supplementary Table S1. Measurement Schedule and Scoring**

| Construct | Questionnaire code | No. of items | Wave(s) | Scoring |
| --- | --- | --- | --- | --- |
| Perceived geopolitical and economic uncertainty | IUP / PGECU | 6 | T1 | Mean of IUP1–IUP6 |
| Future development anxiety | FDA | 6 | T1 and T2 | Mean of FDA1–FDA6 at each wave |
| Academic burnout | AB | 9 | T1 and T2 | Mean of AB1–AB9 at each wave |
| Employment competition pressure | ECP | 6 | T1 | Mean of ECP1–ECP6 |
| Perceived structural university support | USS / PSUS | 8 | T1 | Mean of USS1–USS8 |

**Supplementary Table S2. English Items for Perceived Geopolitical and Economic Uncertainty (PGECU; Questionnaire Code IUP)**

| Code | English item |
| --- | --- |
| IUP1 | Current international developments are changing rapidly, and their future direction is difficult to predict. |
| IUP2 | Changes in international politics, the economy, or conflicts make me worry about my future development. |
| IUP3 | I think instability in the international environment may affect university students’ employment and further-study opportunities. |
| IUP4 | When facing changes in the international situation, I am often uncertain about how to adjust my future plans. |
| IUP5 | I worry that changes in the international environment will make previously clear development pathways unstable. |
| IUP6 | When I encounter information about international developments, I feel that my future development involves more uncontrollable factors. |

**Supplementary Table S3. English Items for Future Development Anxiety (FDA)**

| Code | English item |
| --- | --- |
| FDA1 | Thinking about future employment or further study makes me feel noticeably anxious. |
| FDA2 | I worry that my future development opportunities will become increasingly limited. |
| FDA3 | I often worry that my life after graduation will not meet my expectations. |
| FDA4 | When planning for the future, I often feel confused and uneasy. |
| FDA5 | I worry that my current efforts are insufficient to cope with future competition. |
| FDA6 | Uncertainty about the future affects my current study and daily life. |

**Supplementary Table S4. English Items for Academic Burnout (AB)**

| Code | English item |
| --- | --- |
| AB1 | Recently, studying often makes me feel exhausted. |
| AB2 | Even after resting, I still find it difficult to recover my energy for studying. |
| AB3 | I feel that academic tasks often cause substantial psychological exhaustion. |
| AB4 | My interest in some courses or learning tasks has clearly declined. |
| AB5 | I sometimes feel that studying is merely about completing tasks. |
| AB6 | When facing academic requirements, I am prone to thoughts of avoidance or procrastination. |
| AB7 | I feel that my learning efficiency is lower than before. |
| AB8 | My confidence in achieving my academic goals has declined. |
| AB9 | I find it difficult to gain a sense of accomplishment from studying. |

**Supplementary Table S5. English Items for Employment Competition Pressure (ECP)**

| Code | English item |
| --- | --- |
| ECP1 | I feel that competition for employment among university students is very intense. |
| ECP2 | I worry that I lack sufficient advantages in employment competition. |
| ECP3 | My peers’ job preparation makes me feel pressured. |
| ECP4 | I think employers’ requirements for graduates are becoming increasingly demanding. |
| ECP5 | I worry that the number of desirable jobs available to me in the future will be limited. |
| ECP6 | Employment competition makes me feel more uneasy about my future development. |

**Supplementary Table S6. English Items for Perceived Structural University Support (PSUS; Questionnaire Code USS)**

| Code | English item |
| --- | --- |
| USS1 | The university provides students with clear guidance on academic development. |
| USS2 | When I encounter difficulties in study or development planning, the university provides appropriate channels for assistance. |
| USS3 | The university’s career guidance, recruitment information, or career planning services are practically helpful to me. |
| USS4 | The university communicates information about further study, employment, or development opportunities in a timely manner. |
| USS5 | The university takes students’ needs related to academic pressure, employment pressure, and psychological distress seriously. |
| USS6 | I know that I can obtain appropriate support from the university when I experience psychological stress. |
| USS7 | Support from instructors, counselors, or supervisors at the university is reliable. |
| USS8 | Overall, I believe that the university provides stable support for students’ future development. |

**Appendix 2**

**Supplementary Table S7. Mahalanobis Distance Test**

| **T1** | | | **T2** | | |
| --- | --- | --- | --- | --- | --- |
| **Participant ID** | **Mahalanobis distance** | **p** | **Participant ID** | **Mahalanobis distance** | **p** |
| 1 | 13.120 | 0.010 | 156 | 4.219 | 0.040 |
| 1213 | 12.209 | 0.020 | 75 | 4.219 | 0.040 |
| 1214 | 12.209 | 0.020 | 74 | 4.219 | 0.040 |
| 1215 | 12.209 | 0.020 | 92 | 4.219 | 0.040 |
| 885 | 11.751 | 0.020 | 44 | 4.219 | 0.040 |
| 140 | 11.522 | 0.020 | 1078 | 4.219 | 0.040 |
| 241 | 11.236 | 0.020 | 826 | 4.219 | 0.040 |
| 100 | 10.672 | 0.030 | 301 | 4.219 | 0.040 |
| 70 | 10.498 | 0.030 | 300 | 4.219 | 0.040 |
| 1181 | 10.442 | 0.030 | 493 | 4.219 | 0.040 |

**Supplementary Table S8. Participant Characteristics**

| Variable | Category | Frequency (n) | Percentage (%) |
| --- | --- | --- | --- |
| Gender | Male | 585 | 48.100 |
|  | Female | 630 | 51.900 |
| Year of study | First year | 291 | 24.000 |
|  | Second year | 267 | 22.000 |
|  | Third year | 323 | 26.600 |
|  | Fourth year | 259 | 21.300 |
|  | Postgraduate | 75 | 6.200 |
| Field of study | Humanities | 104 | 8.600 |
|  | Education | 200 | 16.500 |
|  | Economics and management | 244 | 20.100 |
|  | Science and engineering | 135 | 11.100 |
|  | Medicine | 86 | 7.100 |
|  | Arts and sports | 331 | 27.200 |
|  | Other | 115 | 9.500 |
| Type of institution | Comprehensive | 406 | 33.400 |
|  | Teacher education | 213 | 17.500 |
|  | Science and engineering | 293 | 24.100 |
|  | Finance and economics | 147 | 12.100 |
|  | Medicine | 84 | 6.900 |
|  | Other | 72 | 5.900 |
| Primary postgraduation plan | Employment | 397 | 32.700 |
|  | Postgraduate study/further education | 328 | 27.000 |
|  | Civil service/public-sector examination | 216 | 17.800 |
|  | Study abroad/overseas entrepreneurship | 110 | 9.100 |
|  | Temporarily undecided | 54 | 4.400 |
|  | Not yet determined | 110 | 9.100 |
| Self-rated family economic status | Financially difficult | 232 | 19.100 |
|  | Average | 661 | 54.400 |
|  | Good | 277 | 22.800 |
|  | Very good | 45 | 3.700 |
| Self-rated academic performance or GPA | Low | 85 | 7.000 |
|  | Below average | 215 | 17.700 |
|  | Average | 535 | 44.000 |
|  | Above average | 292 | 24.000 |
|  | High | 88 | 7.200 |
| Frequency of following international news or changes in the international situation | Almost never | 156 | 12.800 |
|  | Occasionally | 332 | 27.300 |
|  | Sometimes | 387 | 31.900 |
|  | Often | 274 | 22.600 |
|  | Very often | 66 | 5.400 |
| Frequency of following employment, further-study, or career-development information | Almost never | 112 | 9.200 |
|  | Occasionally | 225 | 18.500 |
|  | Sometimes | 472 | 38.800 |
|  | Often | 307 | 25.300 |
|  | Very often | 99 | 8.100 |
| Major event substantially affecting study or mood during the previous month | No | 934 | 76.900 |
|  | Yes | 281 | 23.100 |

**Supplementary Table S9. Item Descriptive Statistics**

| Item | Mean (M) | SD | Skewness | Kurtosis |
| --- | --- | --- | --- | --- |
| PGECU1 | 3.026 | 1.239 | -0.069 | -1.016 |
| PGECU2 | 3.003 | 1.253 | 0.044 | -1.006 |
| PGECU3 | 3.054 | 1.228 | 0.001 | -0.985 |
| PGECU4 | 3.014 | 1.220 | -0.024 | -0.974 |
| PGECU5 | 3.031 | 1.233 | -0.004 | -0.990 |
| PGECU6 | 3.015 | 1.270 | 0.001 | -1.041 |
| T1_FDA1 | 3.011 | 1.240 | -0.015 | -0.994 |
| T1_FDA2 | 3.021 | 1.241 | -0.013 | -0.993 |
| T1_FDA3 | 3.015 | 1.245 | 0.013 | -0.989 |
| T1_FDA4 | 3.006 | 1.255 | 0.004 | -0.999 |
| T1_FDA5 | 3.019 | 1.258 | -0.031 | -1.026 |
| T1_FDA6 | 2.964 | 1.280 | 0.013 | -1.075 |
| T1_AB1 | 2.998 | 1.227 | 0.027 | -0.961 |
| T1_AB2 | 3.032 | 1.250 | -0.018 | -1.002 |
| T1_AB3 | 2.990 | 1.270 | -0.008 | -1.037 |
| T1_AB4 | 2.996 | 1.260 | 0.070 | -1.038 |
| T1_AB5 | 3.023 | 1.261 | -0.024 | -1.011 |
| T1_AB6 | 3.018 | 1.255 | -0.007 | -1.013 |
| T1_AB7 | 3.007 | 1.249 | -0.017 | -0.995 |
| T1_AB8 | 2.998 | 1.232 | -0.014 | -0.980 |
| T1_AB9 | 3.005 | 1.239 | 0.035 | -0.993 |
| ECP1 | 3.015 | 1.229 | -0.055 | -0.957 |
| ECP2 | 3.058 | 1.253 | 0.003 | -1.012 |
| ECP3 | 3.007 | 1.242 | -0.020 | -1.035 |
| ECP4 | 3.025 | 1.256 | -0.012 | -1.015 |
| ECP5 | 2.998 | 1.210 | -0.051 | -0.913 |
| ECP6 | 3.008 | 1.216 | -0.024 | -0.926 |
| PSUS1 | 2.974 | 1.236 | 0.001 | -0.973 |
| PSUS2 | 2.960 | 1.238 | 0.048 | -0.995 |
| PSUS3 | 3.013 | 1.236 | -0.051 | -0.977 |
| PSUS4 | 2.996 | 1.241 | -0.003 | -0.976 |
| PSUS5 | 3.024 | 1.290 | 0.004 | -1.090 |
| PSUS6 | 2.992 | 1.262 | -0.021 | -1.020 |
| PSUS7 | 3.006 | 1.219 | -0.014 | -0.926 |
| PSUS8 | 3.021 | 1.259 | -0.020 | -0.989 |
| T2_FDA1 | 3.017 | 1.211 | -0.028 | -0.965 |
| T2_FDA2 | 3.028 | 1.231 | -0.016 | -0.953 |
| T2_FDA3 | 3.016 | 1.239 | -0.048 | -0.984 |
| T2_FDA4 | 3.021 | 1.245 | 0.030 | -0.996 |
| T2_FDA5 | 2.990 | 1.211 | 0.011 | -0.960 |
| T2_FDA6 | 3.017 | 1.231 | -0.033 | -0.958 |
| T2_AB1 | 3.023 | 1.249 | -0.003 | -1.026 |
| T2_AB2 | 3.001 | 1.223 | 0.034 | -0.976 |
| T2_AB3 | 2.977 | 1.219 | 0.058 | -0.921 |
| T2_AB4 | 3.017 | 1.251 | 0.018 | -0.995 |
| T2_AB5 | 3.034 | 1.188 | -0.006 | -0.884 |
| T2_AB6 | 3.035 | 1.231 | -0.070 | -0.953 |
| T2_AB7 | 3.000 | 1.230 | -0.027 | -0.937 |
| T2_AB8 | 3.022 | 1.202 | -0.054 | -0.894 |
| T2_AB9 | 2.986 | 1.219 | 0.038 | -0.907 |

**Supplementary Table S10. KMO and Bartlett's Tests**

| Scale | KMO | Bartlett χ² | df | p |
| --- | --- | --- | --- | --- |
| PGECU | 0.918 | 3774.4 | 15 | < 0.001 |
| T1 FDA | 0.912 | 3445.82 | 15 | < 0.001 |
| T1 AB | 0.958 | 5756.66 | 36 | < 0.001 |
| ECP | 0.914 | 3527.79 | 15 | < 0.001 |
| PSUS | 0.948 | 4943.71 | 28 | < 0.001 |
| T2 FDA | 0.912 | 3450.12 | 15 | < 0.001 |
| T2 AB | 0.954 | 5314.12 | 36 | < 0.001 |
| Full scale | 0.966 | 50827.91 | 1225 | < 0.001 |

**Supplementary Table S11. Common Method Bias**

| Component | Initial eigenvalues | | | Extraction sums of squared loadings | | | Rotation sums of squared loadings | | |
| --- | --- | --- | --- | --- | --- | --- | --- | --- | --- |
|  | Total | % of variance | Cumulative % | Total | % of variance | Cumulative % | Total | % of variance | Cumulative % |
| 1 | 16.747 | 33.495 | 33.495 | 16.747 | 33.495 | 33.495 | 7.506 | 15.012 | 15.012 |
| 2 | 5.252 | 10.503 | 43.998 | 5.252 | 10.503 | 43.998 | 6.02 | 12.04 | 27.051 |
| 3 | 3.415 | 6.831 | 50.829 | 3.415 | 6.831 | 50.829 | 5.145 | 10.29 | 37.341 |
| 4 | 3.148 | 6.296 | 57.125 | 3.148 | 6.296 | 57.125 | 4.782 | 9.563 | 46.905 |
| 5 | 3.056 | 6.112 | 63.237 | 3.056 | 6.112 | 63.237 | 4.194 | 8.388 | 55.292 |
| 6 | 2.179 | 4.357 | 67.594 | 2.179 | 4.357 | 67.594 | 4.126 | 8.252 | 63.544 |
| 7 | 1.993 | 3.986 | 71.581 | 1.993 | 3.986 | 71.581 | 4.018 | 8.036 | 71.581 |

**Supplementary Table S12. Rotated Component Matrix**

| Item | 1 | 2 | 3 | 4 | 5 | 6 | 7 |
| --- | --- | --- | --- | --- | --- | --- | --- |
| PGECU1 |  |  |  |  |  |  | 0.756 |
| PGECU2 |  |  |  |  |  |  | 0.751 |
| PGECU3 |  |  |  |  |  |  | 0.742 |
| PGECU4 |  |  |  |  |  |  | 0.752 |
| PGECU5 |  |  |  |  |  |  | 0.716 |
| PGECU6 |  |  |  |  |  |  | 0.721 |
| T1_FDA1 |  |  |  | 0.806 |  |  |  |
| T1_FDA2 |  |  |  | 0.801 |  |  |  |
| T1_FDA3 |  |  |  | 0.815 |  |  |  |
| T1_FDA4 |  |  |  | 0.788 |  |  |  |
| T1_FDA5 |  |  |  | 0.810 |  |  |  |
| T1_FDA6 |  |  |  | 0.808 |  |  |  |
| T1_AB1 |  | 0.761 |  |  |  |  |  |
| T1_AB2 |  | 0.763 |  |  |  |  |  |
| T1_AB3 |  | 0.756 |  |  |  |  |  |
| T1_AB4 |  | 0.736 |  |  |  |  |  |
| T1_AB5 |  | 0.746 |  |  |  |  |  |
| T1_AB6 |  | 0.688 |  |  |  |  |  |
| T1_AB7 |  | 0.717 |  |  |  |  |  |
| T1_AB8 |  | 0.773 |  |  |  |  |  |
| T1_AB9 |  | 0.773 |  |  |  |  |  |
| ECP1 |  |  |  |  | 0.781 |  |  |
| ECP2 |  |  |  |  | 0.802 |  |  |
| ECP3 |  |  |  |  | 0.791 |  |  |
| ECP4 |  |  |  |  | 0.798 |  |  |
| ECP5 |  |  |  |  | 0.795 |  |  |
| ECP6 |  |  |  |  | 0.787 |  |  |
| PSUS1 |  |  | 0.767 |  |  |  |  |
| PSUS2 |  |  | 0.796 |  |  |  |  |
| PSUS3 |  |  | 0.787 |  |  |  |  |
| PSUS4 |  |  | 0.786 |  |  |  |  |
| PSUS5 |  |  | 0.802 |  |  |  |  |
| PSUS6 |  |  | 0.787 |  |  |  |  |
| PSUS7 |  |  | 0.790 |  |  |  |  |
| PSUS8 |  |  | 0.787 |  |  |  |  |
| T2_FDA1 |  |  |  |  |  | 0.730 |  |
| T2_FDA2 |  |  |  |  |  | 0.750 |  |
| T2_FDA3 |  |  |  |  |  | 0.763 |  |
| T2_FDA4 |  |  |  |  |  | 0.755 |  |
| T2_FDA5 |  |  |  |  |  | 0.757 |  |
| T2_FDA6 |  |  |  |  |  | 0.750 |  |
| T2_AB1 | 0.844 |  |  |  |  |  |  |
| T2_AB2 | 0.839 |  |  |  |  |  |  |
| T2_AB3 | 0.840 |  |  |  |  |  |  |
| T2_AB4 | 0.852 |  |  |  |  |  |  |
| T2_AB5 | 0.834 |  |  |  |  |  |  |
| T2_AB6 | 0.835 |  |  |  |  |  |  |
| T2_AB7 | 0.860 |  |  |  |  |  |  |
| T2_AB8 | 0.841 |  |  |  |  |  |  |
| T2_AB9 | 0.851 |  |  |  |  |  |  |

Extraction method: Principal component analysis.

Rotation method: Varimax with Kaiser normalization.

a Rotation converged in six iterations.

**Supplementary Table S13. Reliability Analysis and Q² Effect Sizes**

| Variable | Cronbach's α | rho_A | rho_C | AVE | Q² |
| --- | --- | --- | --- | --- | --- |
| T1 AB | 0.935 | 0.937 | 0.945 | 0.658 | 0.566 |
| T1 FDA | 0.962 | 0.962 | 0.969 | 0.841 | 0.770 |
| T2 AB | 0.977 | 0.977 | 0.98 | 0.847 | 0.799 |
| T2 FDA | 0.903 | 0.904 | 0.925 | 0.672 | 0.537 |
| PGECU | 0.889 | 0.889 | 0.915 | 0.643 | 0.497 |
| ECP | 0.902 | 0.905 | 0.925 | 0.671 | 0.536 |
| PSUS | 0.917 | 0.917 | 0.932 | 0.632 | 0.522 |

**Supplementary Table S14. HTMT Validity Test**

|  | T1 AB | T1 FDA | T2 AB | T2 FDA | PGECU | ECP | PSUS |
| --- | --- | --- | --- | --- | --- | --- | --- |
| T1 AB |  |  |  |  |  |  |  |
| T1 FDA | 0.551 |  |  |  |  |  |  |
| T2 AB | 0.546 | 0.54 |  |  |  |  |  |
| T2 FDA | 0.399 | 0.556 | 0.523 |  |  |  |  |
| PGECU | 0.481 | 0.531 | 0.473 | 0.450 |  |  |  |
| ECP | 0.357 | 0.337 | 0.302 | 0.318 | 0.280 |  |  |
| PSUS | 0.177 | 0.134 | 0.196 | 0.207 | 0.040 | 0.059 |  |

**Supplementary Table S15. Model Factor Loadings and VIF Values**

|  | T1 AB | T1 FDA | T2 AB | T2 FDA | PGECU | ECP | PSUS | VIF |
| --- | --- | --- | --- | --- | --- | --- | --- | --- |
| ECP1 |  |  |  |  |  | 0.821 |  | 2.232 |
| ECP2 |  |  |  |  |  | 0.827 |  | 2.339 |
| ECP3 |  |  |  |  |  | 0.803 |  | 2.238 |
| ECP4 |  |  |  |  |  | 0.812 |  | 2.276 |
| ECP5 |  |  |  |  |  | 0.819 |  | 2.308 |
| ECP6 |  |  |  |  |  | 0.833 |  | 2.352 |
| PGECU1 |  |  |  |  | 0.808 |  |  | 2.122 |
| PGECU2 |  |  |  |  | 0.815 |  |  | 2.158 |
| PGECU3 |  |  |  |  | 0.795 |  |  | 2.06 |
| PGECU4 |  |  |  |  | 0.799 |  |  | 2.086 |
| PGECU5 |  |  |  |  | 0.789 |  |  | 2.029 |
| PGECU6 |  |  |  |  | 0.805 |  |  | 2.116 |
| T1_AB1 | 0.793 |  |  |  |  |  |  | 2.431 |
| T1_AB2 | 0.795 |  |  |  |  |  |  | 2.453 |
| T1_AB3 | 0.797 |  |  |  |  |  |  | 2.378 |
| T1_AB4 | 0.815 |  |  |  |  |  |  | 2.501 |
| T1_AB5 | 0.816 |  |  |  |  |  |  | 2.506 |
| T1_AB6 | 0.829 |  |  |  |  |  |  | 2.666 |
| T1_AB7 | 0.824 |  |  |  |  |  |  | 2.637 |
| T1_AB8 | 0.808 |  |  |  |  |  |  | 2.41 |
| T1_AB9 | 0.821 |  |  |  |  |  |  | 2.465 |
| T1_FDA1 |  | 0.917 |  |  |  |  |  | 4.568 |
| T1_FDA2 |  | 0.913 |  |  |  |  |  | 4.332 |
| T1_FDA3 |  | 0.919 |  |  |  |  |  | 4.578 |
| T1_FDA4 |  | 0.912 |  |  |  |  |  | 4.269 |
| T1_FDA5 |  | 0.922 |  |  |  |  |  | 4.709 |
| T1_FDA6 |  | 0.919 |  |  |  |  |  | 4.505 |
| T2_AB1 |  |  | 0.913 |  |  |  |  | 4.972 |
| T2_AB2 |  |  | 0.918 |  |  |  |  | 5.136 |
| T2_AB3 |  |  | 0.913 |  |  |  |  | 4.886 |
| T2_AB4 |  |  | 0.92 |  |  |  |  | 5.328 |
| T2_AB5 |  |  | 0.917 |  |  |  |  | 5.168 |
| T2_AB6 |  |  | 0.921 |  |  |  |  | 5.603 |
| T2_AB7 |  |  | 0.930 |  |  |  |  | 5.987 |
| T2_AB8 |  |  | 0.921 |  |  |  |  | 5.449 |
| T2_AB9 |  |  | 0.927 |  |  |  |  | 5.729 |
| T2_FDA1 |  |  |  | 0.820 |  |  |  | 2.363 |
| T2_FDA2 |  |  |  | 0.847 |  |  |  | 2.607 |
| T2_FDA3 |  |  |  | 0.807 |  |  |  | 2.275 |
| T2_FDA4 |  |  |  | 0.805 |  |  |  | 2.236 |
| T2_FDA5 |  |  |  | 0.825 |  |  |  | 2.303 |
| T2_FDA6 |  |  |  | 0.815 |  |  |  | 2.218 |
| PSUS1 |  |  |  |  |  |  | 0.783 | 2.131 |
| PSUS2 |  |  |  |  |  |  | 0.802 | 2.26 |
| PSUS3 |  |  |  |  |  |  | 0.785 | 2.125 |
| PSUS4 |  |  |  |  |  |  | 0.794 | 2.166 |
| PSUS5 |  |  |  |  |  |  | 0.801 | 2.24 |
| PSUS6 |  |  |  |  |  |  | 0.799 | 2.187 |
| PSUS7 |  |  |  |  |  |  | 0.799 | 2.155 |
| PSUS8 |  |  |  |  |  |  | 0.796 | 2.156 |

**Supplementary Table S16. Model Fit**

| Variable | R² | Adjusted R² | SRMR | NFI |
| --- | --- | --- | --- | --- |
| T1 AB | 0.196 | 0.195 | 0.028 | 0.929 |
| T1 FDA | 0.242 | 0.241 |  |  |
| T2 AB | 0.455 | 0.446 |  |  |
| T2 FDA | 0.304 | 0.302 |  |  |

**Supplementary Table S17. f² Effect Sizes**

| Path | f² |
| --- | --- |
| T1 AB -> T2 AB | 0.071 |
| T1 AB -> T2 FDA | 0.007 |
| T1 FDA -> T2 AB | 0.031 |
| T1 FDA -> T2 FDA | 0.137 |
| T2 FDA -> T2 AB | 0.054 |
| PGECU -> T1 AB | 0.244 |
| PGECU -> T1 FDA | 0.319 |
| PGECU -> T2 AB | 0.028 |
| PGECU -> T2 FDA | 0.031 |
| ECP -> T2 AB | 0.003 |
| PSUS -> T2 AB | 0.005 |
| ECP × PGECU -> T2 AB | 0.019 |
| PSUS × ECP -> T2 AB | 0 |
| PSUS × ECP × PGECU -> T2 AB | 0.024 |
| PSUS × PGECU -> T2 AB | 0.001 |
